# Fractional Order Dual-Phase-Lag Model of Heat Conduction in a Composite Spherical Medium

**DOI:** 10.3390/ma15207251

**Published:** 2022-10-17

**Authors:** Stanisław Kukla, Urszula Siedlecka, Mariusz Ciesielski

**Affiliations:** 1 Department of Mathematics, Czestochowa University of Technology, Armii Krajowej 21, 42-200 Czestochowa, Poland; 2 Department of Computer Science, Czestochowa University of Technology, Dabrowskiego 73, 42-200 Czestochowa, Poland

**Keywords:** heat transfer, dual phase lag model, fractional Caputo derivative, Laplace transform, composite spherical medium

## Abstract

In the paper, a solution of the fractional dual-phase-lag heat conduction problem is presented. The considerations are related to the heat conduction in a multi-layered spherical medium with azimuthal symmetry. The final form of the analytical solution is given in a form of the double series of spherical Bessel functions and Legendre functions. Numerical calculations concern the study of the effect of the order of the Caputo derivative on the temperature distribution in a composite solid sphere, hemisphere and spherical cone.

## 1. Introduction

Mathematical modeling of the heat conduction is an important stage in the design of systems subjected to thermal loads, because it allows the appropriate selection of materials to avoid adverse phenomena related to the occurrence of thermal stresses. For example, the temperature distribution in the tested system may lead to the formation of thermal stresses, which may cause micro-cracks in the components of this system [1,2,3], and cyclical changes in the thermal load of the device may cause undesirable vibrations of the components of this device [4,5,6]. On the other hand, the heat source causing high temperature of biological tissue may destroy diseased tissue, but it may also damage healthy tissue [7,8]. Different mathematical models are used to accurately describe the heat conduction in the considered bodies.

The classical mathematical model of heat conduction is derived from Fourier’s law of the heat conduction. The Fourier law establishes a proportionality between the heat flux vector and the temperature gradient [9]

(1) $$q\left(r,t\right)=-\lambda \;\nabla T\left(r,t\right),$$

where q is the heat flux vector, λ is the thermal conductivity of the material, r is the point in the considered region, t is the time, ∇ is the gradient operator and T is the temperature. Although the Fourier law quite accurately describes the heat conduction in most practical macroscopic problems, the relationship (1) implies an unrealistic infinite speed of the heat propagation; this means that the sudden temperature change at some point in the domain will be felt everywhere and instantaneously at distant points in the domain—hence, Fourier’s law can be treated as having the unphysical property [10]. To eliminate this drawback of the mathematical model, the Fourier law (1) is replaced by the following relationship [11]

(2) $$q\left(r,t+\tau_{q}\right)=-\lambda \nabla T\left(r,t+\tau_{T}\right),$$

where $\tau_{q}$ and $\tau_{T}$ are the phase lags. Relationship (2) for $\tau_{q}>0$ and $\tau_{T}>0$ is the dual-phase-lag constitutive equation for the heat conduction. The introduction of these phase lags into this model is interpreted as the relaxation times accounting for the effects of thermal inertia. The left and right sides of Equation (2) are expanded into the Taylor series. Depending on the assumed number of the terms of this series, the different forms of the dual-phase-lag model can be obtained. In Ref. [10], the authors discussed different forms of the dual-phase-lag model as well as presented their characteristics. In this work, the first-order approximation of the functions occurring on the left- and right-hand side of Equation (2) is considered, which leads to the constitutive equation for the heat conduction in the form

(3) $$q\left(r,t\right)+\tau_{q}\frac{\partial q\left(r,t\right)}{\partial t}=-\lambda \left(\nabla T\left(r,t\right)+\tau_{T}\frac{\partial }{\partial t}\nabla T\left(r,t\right)\right)\;.$$

In addition, the energy equation is introduced [12]

(4) $$-\nabla \cdot q\left(r,t\right)+g\left(r,t\right)=\rho C_{p}\frac{\partial T\left(r,t\right)}{\partial t},$$

where $g\left(r,t\right)$ is the volumetric rate of the heat generation, ρ is the density of the material and $C_{p}$ is the specific heat capacity. Eliminating the heat flux from Equations (3) and (4), we obtain the heat conduction equation in the form

(5) $$\frac{\tau_{q}}{\kappa }\frac{\partial^{2}T\left(r,t\right)}{\partial t^{2}}+\frac{1}{\kappa }\frac{\partial T\left(r,t\right)}{\partial t}=\tau_{T}\frac{\partial }{\partial t}\nabla^{2}T\left(r,t\right)+\nabla^{2}T\left(r,t\right)+\frac{\tau_{q}}{\lambda }\frac{\partial g\left(r,t\right)}{\partial t}+\frac{1}{\lambda }g\left(r,t\right),$$

where $\nabla^{2}$ is the Laplace operator and κ=λλρCpρCp is the thermal diffusivity.

The dual-phase-lag heat shown in Equation (5) has been applied in mathematical modeling of the heat transfer in functionally graded materials [13,14,15], ultrafast pulse-laser heating problems [16,17], porous media [18,19,20], nanocomposites [21,22], and living tissue [23,24,25]. If $\tau_{q}=\tau_{T}=0$ in Equation (5), then the classical parabolic heat conduction equation is obtained. For $\tau_{T}=0$ and $\tau_{q}>0$, one obtains the single-phase-lag equation of hyperbolic type. The wave character of this heat conduction equation was used in the investigations of propagations of heat waves in papers [26,27].

A generalization of the dual-phase-lag model of the heat conduction is obtained by replacement of the derivatives in the constitutive equation and energy equation by the derivatives of non-integer order. In the present paper, the Caputo derivative of non-integer order α is used and is defined by [28]

(6) Dtα0Cft≡dαftdtα=1Γm−α∫0tt−τm−α−1dmfτdτmdτ,m−1<α<m.

The properties of the derivative can be found in many books devoted to fractional calculus, for instance in the books [28,29,30,31].

Replacing the time derivatives in the constitutive Equation (3) and energy Equation (4) by the Caputo time fractional derivatives, one obtains

(7) $$q\left(r,t\right)+\tau_{q}\nu^{\alpha -1}\frac{\partial^{\alpha }q\left(r,t\right)}{\partial t^{\alpha }}=-\lambda \left(\nabla T\left(r,t\right)+\tau_{T}\nu^{\alpha -1}\frac{\partial^{\alpha }}{\partial t^{\alpha }}\nabla T\left(r,t\right)\right)\;,$$

(8) $$-\nabla \cdot q\left(r,t\right)+g\left(r,t\right)=\rho C_{p}\nu^{\alpha -1}\frac{\partial^{\alpha }T\left(r,t\right)}{\partial t^{\alpha }},\;\;\;\;\;\;\;0<\alpha \leq 1,$$

where the coefficient ν is introduced to keep the accordance of dimensions. Combining Equations (7) and (8), one obtains the heat conduction equation with the Caputo fractional derivatives in the form

(9) $$\begin{array}{cc} \frac{\partial^{\alpha }}{\partial \;t^{\alpha }}\left(\frac{\partial^{\alpha }}{\partial \;t^{\alpha }}+\frac{1}{\tau_{q}\nu^{\alpha -1}}\right)T\left(r,t\right)= & \frac{\kappa \tau_{T}}{\tau_{q}\nu^{\alpha -1}}\left(\frac{\partial^{\alpha }}{\partial \;t^{\alpha }}+\frac{1}{\tau_{T}\nu^{\alpha -1}}\right)\nabla^{2}T\left(r,t\right)\\ \; & +\frac{\kappa }{\lambda \nu^{\alpha -1}}\left(\frac{\partial^{\alpha }}{\partial \;t^{\alpha }}+\frac{1}{\tau_{q}\nu^{\alpha -1}}\right)g\left(r,t\right). \end{array}$$

We use this equation for modeling the heat conduction in a multi-layered body. According to the authors’ knowledge, this approach to the problem of two-dimensional time-fractional heat conduction in a multi-layered medium was not used in the literature. Due to the definition of the Caputo derivative, we can note that the differential Equation (9) describes the temperature distribution in a body, taking into account the history of changes of the temperature.

The fractional Equation (9) describes the heat conduction in a region which is specified by the considered medium. In this paper, we consider the fractional heat conduction in a spherical body. The Laplace operator which occurs in Equation (9) in the spherical co-ordinates is defined as follows

(10) $$\nabla^{2}=\frac{1}{r^{2}}\left[\frac{\partial }{\partial r}\left(r^{2}\frac{\partial }{\partial r}\right)+\frac{1}{\sin\varphi }\frac{\partial }{\partial \varphi }\left(\sin\varphi \frac{\partial }{\partial \varphi }\right)+\frac{1}{\sin^{2}\varphi }\frac{\partial^{2}}{\partial \theta^{2}}\right],$$

where r is the radial coordinate, φ is the polar and θ is the azimuthal angle coordinate. Assuming that the temperature distribution in the body is azimuthally invariant (azimuthal symmetry), the last term in the bracket in Equation (10) can be dropped. A simplified form of the Laplace operator can be obtained by introducing a new variable μ which is related to the polar angle φ by the relationship

(11) $$\mu =\cos\left(\varphi \right)\;.$$

Taking into account the azimuthal symmetry, the Laplace operator in the new coordinates can be written in the form

(12) $$\nabla^{2}=\frac{1}{r^{2}}\left[\frac{\partial }{\partial r}\left(r^{2}\frac{\partial }{\partial r}\right)+\frac{\partial }{\partial \mu }\left(\left(1-\mu^{2}\right)\frac{\partial }{\partial \mu }\right)\right]\;.$$

In this paper, we present a dual-phase-lag fractional heat conduction model for a multi-layered spherical body with azimuthal symmetry. An analytical solution of the problem is derived in the form of the double series of spherical Bessel functions and Legendre functions. Numerical computations of the temperature distribution in the body include a composite solid sphere, hemisphere and spherical cone. Effects of the fractional order of time-derivatives and phase lagging occurring in the heat conduction model on the temperature distribution in the considered bodies are investigated numerically.

The problem considered in this paper is a continuation of the research that has been presented in papers [32,33,34,35,36]. A solution of the fractional heat conduction problem in the solid sphere was presented in [32]. The authors studied a fractional single-phase lag heat conduction problem in the whole-space 1D domain [34], in the slab [35] and in the hollow cylinder [33]. A fractional dual-phase-lagging heat conduction in the whole-space 1D domain was presented in [36].

## 2. Formulation of the Problem

**Figure 1:**
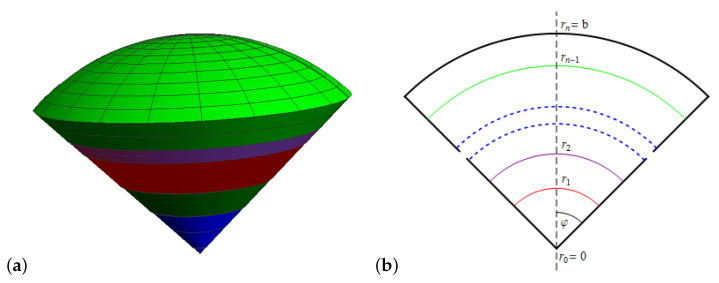
Multi-layered spherical cone under considerations: (**a**) 3D graph, (**b**) main cross-section.

Let us consider the fractional heat conduction in a spherical body (with coordinates $r\equiv \left\{r,\varphi \right\}$ consisting of n concentric spherical layers which are defined by: $r_{i-1}⩽r⩽r_{i}$ (i=1,…,n), $0⩽\varphi ⩽\bar{\varphi }$, where $0<\bar{\varphi }⩽\pi$. The body is a full solid sphere when $\bar{\varphi }$ tends to π, for φ¯=ππ22, the body is a hemisphere, and for $0<\bar{\varphi }<\pi /2$, the body is a spherical cone (Figure 1).

The heat conduction in the i-th spherical layer is governed by the following time-fractional differential equation

(13) $$\frac{\partial^{\alpha }}{\partial t^{\alpha }}\left(\frac{\partial^{\alpha }}{\partial t^{\alpha }}+\frac{1}{\tau_{q}\nu^{\alpha -1}}\right)T_{i}=\frac{\kappa_{i}\tau_{T}}{\tau_{q}\nu^{\alpha -1}}\left(\frac{\partial^{\alpha }}{\partial t^{\alpha }}+\frac{1}{\tau_{T}\nu^{\alpha -1}}\right)\nabla^{2}T_{i}+\frac{\kappa_{i}}{\lambda_{i}\nu^{\alpha -1}}\left(\frac{\partial^{\alpha }}{\partial t^{\alpha }}+\frac{1}{\tau_{q}\nu^{\alpha -1}}\right)g_{i},$$

where $T_{i}\equiv T_{i}\left(r,\mu ,t\right)$ is the temperature, $\lambda_{i}$ is the thermal conductivity, $\kappa_{i}$ is the thermal diffusivity, $g_{i}\equiv g_{i}\left(r,\mu ,t\right)$ is the volumetric heat source in the i-th layer and $\alpha \in \left(0,1\right]$ denotes the fractional order of the Caputo derivative, $\mu \in \left[\mu_{\varphi },1\right]$ and $\mu_{\varphi }=\cos\left(\bar{\varphi }\right)$.

The fractional differential Equation (13) for i=1,…,n is complemented by boundary and initial conditions and the conditions providing the perfect thermal contact of the neighboring layers. The conditions are as follows:

(14) $$\left|T_{1}\left(0,\mu ,t\right)\right|<\infty ,$$

(15) $${\left.\frac{\partial T_{i}\left(r,\mu ,t\right)}{\partial \mu }\right|}_{\mu =\mu_{\varphi }}=0,$$

(16) $$T_{i}\left(r_{i},\mu ,t\right)=T_{i+1}\left(r_{i},\mu ,t\right)\;,\;\;\;\;\;\;i=1,\ldots ,n-1,$$

(17) $$\lambda_{i}{\left.\frac{\partial T_{i}\left(r,\mu ,t\right)}{\partial r}\right|}_{r=r_{i}}=\lambda_{i+1}{\left.\frac{\partial T_{i+1}\left(r,\mu ,t\right)}{\partial r}\right|}_{r=r_{i}},\;\;\;\;\;\;\;\;\;\;\;i=1,\ldots ,n-1,$$

(18) $$\lambda_{n}{\left.\frac{\partial T_{n}\left(r,\mu ,t\right)}{\partial r}\right|}_{r=r_{n}}=a_{\infty }\left(T_{\infty }\left(\mu \right)-T_{n}\left(r_{n},\mu ,t\right)\right),$$

where $a_{\infty }$ and $T_{\infty }$ are the outer heat transfer coefficient and ambient temperature, respectively. It should be noted that in general, the boundary conditions (17) and (18) should be formulated using the dual-phase-lag model [37,38]. However, if values of the relaxation times are identical in two neighboring layers, than we can assume the simplification in the above boundary conditions. The initial conditions are assumed in the form

(19) $$T_{i}\left(r,\mu ,0\right)=F_{i}\left(r,\mu \right)\;,\;\;\;\;\;i=1,\ldots ,n,$$

(20) $${\left.\frac{\partial^{\alpha }T_{i}\left(r,\mu ,t\right)}{\partial \;t^{\alpha }}\right|}_{t=0}=G_{i}\left(r,\mu \right),\;\;\;\;\;\;\;i=1,\ldots ,n.$$

## 3. Solution of the Problem

An analytical solution to the initial-boundary problem (13)–(20) can be presented in the form of a sum

(21) $$T_{i}\left(r,\mu ,t\right)=\phi_{i}\left(r,\mu ,t\right)+\psi_{i}\left(r,\mu \right)\;,\;\;\;\;\;i=1,\ldots ,n,$$

where the function $\phi_{i}$ satisfies the fractional differential Equation (13) and homogenous boundary conditions (transient problem) and $\psi_{i}$ is a solution of a steady-state problem. Substituting the function $T_{i}$ in the form (21) into Equation (13), we receive differential equations for the functions $\phi_{i}$ and $\psi_{i}$. For the function $\phi_{i}$, one obtains an equation with fractional time derivative

(22) $$\frac{\partial^{\alpha }}{\partial t^{\alpha }}\left(\frac{\partial^{\alpha }}{\partial t^{\alpha }}+\frac{1}{\tau_{q}\nu^{\alpha -1}}\right)\phi_{i}=\frac{\kappa_{i}\tau_{T}}{\tau_{q}\nu^{\alpha -1}}\left(\frac{\partial^{\alpha }}{\partial t^{\alpha }}+\frac{1}{\tau_{T}\nu^{\alpha -1}}\right)\nabla^{2}\phi_{i}+\frac{\kappa_{i}}{\lambda_{i}\;\nu^{\alpha -1}}\left(\frac{\partial^{\alpha }}{\partial t^{\alpha }}+\frac{1}{\tau_{q}\nu^{\alpha -1}}\right)g_{i},$$

and for the function $\psi_{i}$, one obtains the Laplace equation

(23) $$\nabla^{2}\psi_{i}\left(r,\mu \right)=0.$$

Suppose that $\phi_{i}$ is a function of the form

(24) $$\phi_{i}\left(r,\mu ,t\right)=\Lambda_{\;i}\left(r,\mu \right)\;\theta \left(t\right).$$

Taking into account Equation (24) in the homogenous differential equation obtained by omitting the last term in Equation (22) and separating the space and time variables, we receive the Helmholtz equation for the function $\Lambda_{i}$

(25) $$\nabla^{2}\Lambda_{i}\left(r,\mu \right)+\Omega_{i}^{2}\Lambda_{i}\left(r,\mu \right)=0,$$

where Ωi=ωωκiκi, whereas ω is the separation constant.

In turn, introducing functions $M\left(\mu \right)$, $R_{i}^{\Lambda }\left(r\right)$, $R_{i}^{\psi }\left(r\right)$, we write the functions $\psi_{i}$ and $\Lambda_{i}$ as

(26) $$\psi_{i}\left(r,\;\mu \right)=R_{i}^{\psi }\left(r\right)M\left(\mu \right),\;\;\;\;\Lambda_{i}\left(r,\;\mu \right)=R_{i}^{\Lambda }\left(r\right)\;M\left(\mu \right).$$

Substituting the functions $\psi_{i}$ and $\Lambda_{\;i}$ into Equations (23) and (25), separating the variables and assuming the separation constant as $\beta \left(\beta +1\right)$ where β is a real number, one obtains the three homogenous differential equations: Lagrange equation, Euler equation and spherical Bessel equation:

(27) $$\frac{d}{d\mu }\left(\left(1-\mu^{2}\right)\frac{d}{d\mu }\right)\;M\left(\mu \right)+\beta \left(\beta +1\right)M\left(\mu \right)=0,\;\;\;\mu_{\varphi }⩽\mu ⩽1,$$

(28) $$\frac{d}{dr}\left(r^{2}\frac{d}{dr}\right)R_{i}^{\psi }\left(r\right)-\beta \left(\beta +1\right)R_{i}^{\psi }\left(r\right)=0,\;\;\;r_{i-1}⩽r⩽r_{i},\;\;\;i=1,\ldots ,n,$$

(29) $$\frac{1}{r^{2}}\;\frac{d}{dr}\left(r^{2}\frac{d}{dr}\right)R_{i}^{\Lambda }\left(r\right)+\left(\Omega_{i}^{2}-\frac{\beta \left(\beta +1\right)}{r^{2}}\right)R_{i}^{\Lambda }\left(r\right)=0,\;\;r_{i-1}⩽r⩽r_{i},\;\;\;i=1,\ldots ,n.$$

The functions M, $R_{i}^{\Lambda }$, and $R_{i}^{\psi }$ satisfy boundary conditions which are obtained by taking the functions (21), (24) and (26) in conditions (14)–(18). The function M satisfies the conditions:

(30) $$\left|M\left(\mu \right)\right|<\infty ,\;\;\;\;\mu \in \left[\mu_{\varphi },1\right),$$

(31) $$M^{\prime }\left(\mu_{\varphi }\right)=0.$$

The functions $R_{i}^{\Lambda }\left(r\right)$, $R_{i}^{\psi }\left(r\right)$ satisfy the same conditions at r=0 and at interfaces $r=r_{i}$, i=1,2,…,n−1 (the superscripts are omitted):

(32) $$\left|R_{1}\left(0\right)\right|<\infty ,$$

(33) $$R_{i}\left(r_{i}\right)=R_{i+1}\left(r_{i}\right)\;,\;\;\;\frac{dR_{i}\left(r_{i}\right)}{dr}=\vartheta_{i}\frac{dR_{i+1}\left(r_{i}\right)}{dr}\;,\;\;\;\;\;i=1,\ldots ,n-1,$$

where $\vartheta_{i}=\frac{\lambda_{i+1}}{\lambda_{i}}$. Moreover, the function $R_{n}^{\Lambda }\left(r\right)$ satisfies the homogenous condition at $r=r_{n}$

(34) $$\frac{dR_{n}^{\Lambda }\left(r_{n}\right)}{dr}=-\frac{a_{\infty }}{\lambda_{n}}R_{n}^{\Lambda }\left(r_{n}\right),$$

and the function $R_{\;n}^{\psi }\left(r\right)$, as the radial part of the function $\psi_{n}\left(r,\;\mu \right)=R_{\;n}^{\psi }\left(r\right)\;M\left(\mu \right)$, satisfies the condition

(35) $$\frac{\partial \psi_{n}\left(r_{n},\mu \right)}{\partial r}=\frac{a_{\infty }}{\lambda_{n}}\left(T_{\infty }\left(\mu \right)-\psi_{n}\left(r_{n},\mu \right)\right).$$

The solutions of Equations (27)–(29) with appropriate conditions among (30)–(35) are presented in Section 3.1.

### 3.1. Solution of the Lagrange Equation

The solution to Equation (27), which satisfies the condition (30), is the function

(36) $$M\left(\mu \right)=A_{1}\;P_{\beta }\left(\mu \right),$$

where $P_{\beta }\left(\mu \right)$ is the Legendre function of the first kind and $A_{1}$ is a constant. Using the derivative of the Legendre function [39]

(37) $$\frac{dP_{\beta }\left(\mu \right)}{d\mu }=\frac{\beta +1}{1-\mu^{2}}\left(\mu P_{\beta }\left(\mu \right)-P_{\beta +1}\left(\mu \right)\right),$$

and the boundary condition (31), one obtains the following equation

(38) $$\mu_{\varphi }P_{\beta }\left(\mu_{\varphi }\right)-P_{\beta +1}\left(\mu_{\varphi }\right)=0.$$

The roots of this equation for $\mu_{\varphi }=-1$ (solid sphere) are $\beta_{m}=m$, m=0,1,2,… and for $\mu_{\varphi }=0$ (hemisphere) are $\beta_{m}=2m$, m=0,1,2,…. In these cases, the eigenfunctions $P_{m}\left(\mu \right)$ and $P_{2m}\left(\mu \right)$ where m is a positive integer number are the Legendre polynomials. The roots to Equation (38) for $\mu_{\varphi }\in \left(-1,0\right)\cup \left(0,1\right)$ are numerically determined.

The functions $P_{\beta_{m}}\left(\mu \right)$, m=0,1,2,…, create an orthogonal set of functions. The orthogonality condition of the functions can be derived using an indefinite integral of the product of functions $P_{\beta }\left(\mu \right)$ and $P_{\beta^{\prime }}\left(\mu \right)$ where $\beta \neq \beta^{\prime }$. Utilizing Equation (27), this integral can be expressed as follows

(39) $$\int P_{\beta }\left(\mu \right)P_{\beta^{\prime }}\left(\mu \right)d\mu =\frac{1-\mu^{2}}{\left(\beta -\beta^{\prime }\right)\left(\beta +\beta^{\prime }+1\right)}\left(P_{\beta }\left(\mu \right)\frac{dP_{\beta^{\prime }}\left(\mu \right)}{d\mu }-P_{\beta^{\prime }}\left(\mu \right)\frac{dP_{\beta }\left(\mu \right)}{d\mu }\right)+C,$$

where C is an arbitrary constant. Taking into account the antiderivative given by Equation (39) and the boundary condition (31), one obtains

(40) $$\int_{\mu_{\varphi }}^{1}P_{\beta }\left(\mu \right)P_{\beta^{\prime }}\left(\mu \right)\;d\mu =0\;\;\mathrm{for}\;\;\beta^{\prime }\neq \beta .$$

In order to find the antiderivative of the square of the Legendre function, the integral occurring in Equation (39), employing Equation (37), is rewritten in the form

(41) $$\begin{array}{cc} \; & \int P_{\beta }\left(\mu \right)P_{\beta^{\prime }}\left(\mu \right)d\mu \\ & =\frac{1}{\beta +\beta^{\prime }+1}\left(\frac{\left(\beta^{\prime }+1\right)P_{\beta }\left(\mu \right)P_{\beta^{\prime }+1}\left(\mu \right)-\left(\beta +1\right)P_{\beta +1}\left(\mu \right)P_{\beta^{\prime }}\left(\mu \right)}{\beta^{\prime }-\beta }-\mu P_{\beta }\left(\mu \right)P_{\beta^{\prime }}\left(\mu \right)\right)+C. \end{array}$$

Evaluating limits of the expressions occurring on the left and right-hand sides of Equation (41) as $\beta^{\prime }$ tends to β, the square of the norm $N_{m}^{\mu }$ of the Legendre function for m=1,2,…, one obtains in the form

(42) $$\begin{array}{cc} N_{m}^{\mu } & =\int_{\mu_{\varphi }}^{1}{\left(P_{\beta_{m}}\left(\mu \right)\right)}^{2}d\mu \\ \; & =\frac{\beta_{m}+1}{2\beta_{m}+1}\left(P_{\beta_{m}+1}\left(\mu_{\varphi }\right){\left.\frac{dP_{\beta }\left(\mu_{\varphi }\right)}{d\beta }\right|}_{\beta =\beta_{m}}-P_{\beta_{m}}\left(\mu_{\varphi }\right){\left.\frac{dP_{\beta +1}\left(\mu_{\varphi }\right)}{d\beta }\right|}_{\beta =\beta_{m}}\right), \end{array}$$

where $\beta_{m}$ are the roots of the Equation (38). For m=0, we have $N_{0}^{\mu }=1-\mu_{\varphi }$. Finally, the orthogonality condition of the functions $P_{\beta_{m}}\left(\mu \right)$, m=0,1,2,…, can be written as

(43) $$\int_{\mu_{\varphi }}^{1}P_{\beta_{m}}\left(\mu \right)P_{\beta_{n}}\left(\mu \right)\;d\mu =N_{m}^{\mu }\;\delta_{m\;n},\;\;\mathrm{for}\;\;m,n=0,1,2,\ldots ,$$

where $\delta_{m\;n}$ is the Kronecker delta.

### 3.2. Solution of the Euler Equation in the Multi-Layered Spherical Region

The general solution to the Euler Equation (28) for $\beta =\beta_{m}$, m=0,1,2,…, is given by

(44) $$R_{i,\;m}^{\psi }\left(r\right)=B_{1\;i}{\left(\frac{r}{r_{i}}\right)}^{\beta_{m}}+B_{2\;i}{\left(\frac{r}{r_{i}}\right)}^{-\left(\beta_{m}+1\right)},\;\;r_{i-1}⩽r⩽r_{i},\;\;\;\;i=1,\ldots ,n,$$

where $B_{1\;i}$, $B_{2\;i}$ are arbitrary constants. Using boundary conditions (32), one obtains a set of 2n−2 equations with unknowns: $B_{1\;i}$, $B_{2\;i}$, i=1,…,n−1. These equations are as follows

(45) $$B_{1,\;i}+B_{2,\;i}-\;d_{i}^{\beta_{m}}B_{1,\;i+1}-d_{i}^{-\left(\beta_{m}+1\right)}B_{2,\;i+1}=0,$$

(46) $$B_{1\;,i}-B_{2,\;i}\left(1+\frac{1}{\beta_{m}}\right)-B_{1\;,i+1}\vartheta_{i}d_{i}^{\beta_{m}}+B_{2\;,i+1}\vartheta_{i}\left(1+\frac{1}{\beta_{m}}\right)d_{i}^{-\left(\beta_{m}+1\right)}=0,$$

where $d_{i}=\frac{r_{i}}{r_{i+1}}$. Due to the condition (32), we assume $B_{2\;1}=0$ in Equations (44)–(46).

The system of Equations (45) and (46) is complemented by an equation which is obtained on the basis of the boundary condition (35) for the function $\psi_{n}$. The functions $\psi_{i}$ are given by Equation (26) as a product of two functions. To fulfil the condition (35), we assume that

(47) $$\psi_{i}\left(r,\;\mu \right)=\sum_{m=0}^{\infty }R_{i,m}^{\psi }\left(r\right)\;P_{\beta_{m}}\left(\mu \right),\;\;\;i=1,2,\ldots ,n.$$

Substituting the function $\psi_{n}$ into the condition (35), multiplying the received equation by $P_{\beta_{m^{\prime }}}\left(\mu \right)$, integrating with respect to μ in the interval $\left[\mu_{\varphi },1\right]$ and using the orthogonality condition (43), one obtains the condition for the function $R_{i,m}^{\psi }\left(r\right)$

(48) $$\frac{dR_{n,m}^{\psi }\left(r_{n}\right)}{dr}+\frac{a_{\infty }}{\lambda_{n}}R_{n,m}^{\psi }\left(r_{n}\right)=\frac{a_{\infty }}{N_{m}^{\mu }\lambda_{n}}\int_{\mu_{\varphi }}^{1}T_{\infty }\left(\mu \right)P_{\beta_{m}}\left(\mu \right)\;d\mu .$$

Employing Equation (44) for i=n in Equation (48), the following condition is obtained

(49) $$\left(1+\frac{\beta_{m}\;\lambda_{n}}{a_{\infty }\;r_{n}}\right)B_{1,\;n}+\left(1-\frac{\left(\beta_{m}+1\right)\;\lambda_{n}}{a_{\infty }\;r_{n}}\right)B_{2,\;n}=\frac{I_{m}}{N_{m}^{\mu }},$$

where $I_{m}=\int_{\mu_{\varphi }}^{1}T_{\infty }\left(\mu \right)P_{\beta_{m}}\left(\mu \right)d\mu$. For the function $T_{\infty }$ defined by

(50) $$T_{\infty }\left(\mu \right)=\left\{\begin{array}{cc} 0, & \;\mu_{\varphi }\leq \mu \leq \mu_{0},\\ \left(\mu -\mu_{0}\right)T_{a}, & \;\mu_{0}<\mu \leq 1, \end{array}\right.$$

the integral $I_{m}$ may be expressed in an analytical form as

(51) $$I_{m}=\left\{\begin{array}{cc} \frac{1}{2}{\left(1-\mu_{0}\right)}^{2}T_{a} & \;\mathrm{for}\;\;m=0,\\ \frac{\left(\beta_{m}\left(\mu_{0}^{2}-1\right)+2\mu_{0}^{2}\right)P_{\beta_{m}}\left(\mu_{0}\right)-2\mu_{0}P_{\beta_{m}+1}\left(\mu_{0}\right)}{\beta_{m}\left(\beta_{m}^{2}+\beta_{m}-2\right)}T_{a} & \;\mathrm{for}\;\;m=1,\;2,\;\ldots . \end{array}\right.$$

Equation (49) together with Equations (45) and (46) form a system of 2n−1 equations which is solved for $\beta =\beta_{m}$, m=0,1,2,… with respect to $B_{1\;,1}$, $B_{1\;,2}$, $B_{2,2}$, …, $B_{1\;n}$, $B_{2\;n}$. Hence, the functions $\psi_{i}$, i=1,…,n are now fully defined by Equation (47), whereas the functions $R_{i\;m}^{\psi }$ are given by Equation (44).

### 3.3. Solution of the Spherical Bessel Equation in the Multi-Layered Region

The general solution to Equation (29) can be written as follows

(52) $$\begin{array}{cc} R_{i,m}^{\Lambda }\left(r\right)=C_{1,\;i}\;j_{\beta_{m}}\left(\Omega_{i,m}\;r\right)+C_{2,\;i}\;y_{\beta_{m}}\left(\Omega_{i,m}\;r\right),\;\; & \mathrm{for}\;\;r_{i-1}\leq r\leq r_{i},\\ \; & i=1,\ldots ,n,\;m=0,\;1,\;2,\;\ldots , \end{array}$$

where Ωi,m=ωmωmκiκi, $C_{1,\;i}$, $C_{2,\;i}$ are constants, and $j_{\beta_{m}}$ and $y_{\beta_{m}}$ are spherical Bessel functions of the first and second kind, respectively. The spherical Bessel functions are defined by (see Ref. [9])

(53) jβz=π2zJβ+12z,yβz=π2zYβ+12z,

where $J_{\beta }$ and $Y_{\beta }$ are the Bessel functions of the first and second kind, respectively. The functions $R_{i,m}^{\Lambda }\left(r\right)$ are defined for $r\in \left[r_{i-1},\;r_{i}\right],\;\;\;i=1,2,\ldots ,n$, wherein $r_{0}=0$. As the function $y_{\beta }\left(z\right)$ tends to infinity when z tends to zero, i.e., $\lim_{z\to 0}y_{\beta }\left(z\right)=-\infty$, then taking into account the condition (14), we assume $C_{2,\;1}=0$ in Equation (51) for i=1.

Substituting the function $R_{i,m}^{\Lambda }$ into the continuity conditions (33) and the boundary condition (34), we obtain a system of 2n−1 homogenous equations with unknown $C_{1,\;1}$, $C_{1,2}$, $C_{2,\;2}$, …, $C_{n,\;1}$, $C_{n,\;2}$. This system of equations in the matrix form can be written as

(54) A·C=0,

where $C={\left[C_{1,1}\;\;C_{1,2}\;\;C_{2,2}\;\ldots \;C_{1,n}\;\;C_{2,n}\right]}^{T}$ is the column matrix of the unknowns and $A={\left[a_{ij}\right]}_{1\;⩽\;i,j\;⩽\;2n-1}$ is the coefficients matrix of the equations system. The non-trivial solution of Equation (54) exists if the characteristic equation is satisfied

(55) $$\det\left(A\right)=0.$$

Next, this equation is solved numerically with respect to ω for $\beta =\beta_{m}$, m=0,1,2…. Next, for the roots $\omega_{j,\;m}$, j=1,2,…,m=0,1,2,… of Equation (55), the coefficients $C_{1,2},\;C_{2,2},\ldots ,C_{1,n},\;C_{2,n}$ (index m is omitted) are successfully determined as a solution of Equation (54) with $C_{1,1}=1$. Taking into account the eigenvalues $\omega_{j,\;m}$ and the coefficients $C_{1,i},\;C_{2,i}$ in Equation (51), we obtain the functions $R_{i,\;j,\;m}^{\Lambda }$. These functions satisfy the orthogonality condition which can be derived using the differential Equation (29), the conditions at the interfaces (33) and the boundary condition (34). The orthogonality condition has the form

(56) $$\sum_{i=1}^{n}\frac{\lambda_{i}}{\kappa_{i}}\int_{r_{i-1}}^{r_{i}}r^{2}R_{i,\;j,\;m}^{\Lambda }\left(r\right)\;R_{i,\;j^{\prime },\;m}^{\Lambda }\left(r\right)dr=N_{j,m}^{r}\delta_{j,j^{\prime }},\;\;\;j,\;j^{\prime }=1,\;2\;\ldots ,\;\;m=0,\;1,\;2\;\ldots ,$$

where

(57) $$N_{j,m}^{r}=\sum_{i=1}^{n}\frac{\lambda_{i}}{\kappa_{i}}\int_{r_{i-1}}^{r_{i}}r^{2}{\left(C_{1,\;i}\;j_{\beta_{m}}\left(\Omega_{i,j,m}\;r\right)+C_{2,\;i}\;y_{\beta_{m}}\left(\Omega_{i,j,m}\;r\right)\right)}^{2}\;dr.$$

The functions $\Lambda_{i}$, defined by Equation (26), can be now written as

(58) $$\Lambda_{i,\;j,\;m}\left(r,\mu \right)=R_{i,\;j,m}^{\Lambda }\left(r\right)P_{\beta_{m}}\left(\mu \right).$$

### 3.4. Solution of the Time-Fractional Differential Equation

The functions $\Lambda_{i,\;j,\;m}$ are used to express the functions $\phi_{i}$ in the double series form

(59) $$\phi_{i}\left(r,\mu ,t\right)=\sum_{j=1}^{\infty }\sum_{m=0}^{\infty }\theta_{j,\;m}\left(t\right)\;\Lambda_{i,\;j,\;m}\left(r,\mu \right),$$

where the functions $\theta_{j,\;m}$ satisfy the nonhomogeneous Equation (22). Substituting the functions (59) into Equation (22) and using the orthogonality conditions (43) and (56), we receive the fractional differential equation for the functions $\theta_{j,\;m}$ in the form

(60) $$\begin{array}{cc} \; & \frac{d^{\alpha }}{dt^{\alpha }}\left(\frac{d^{\alpha }\theta_{j,\;m}\left(t\right)}{dt^{\alpha }}\right)+\frac{1}{\tau_{q}\nu^{\alpha -1}}\left(1+\tau_{T}\omega_{j,\;m}^{2}\right)\frac{d^{\alpha }\theta_{j,\;m}\left(t\right)}{dt^{\alpha }}+\frac{\omega_{j,\;m}^{2}}{\tau_{q}\nu^{2\alpha -2}}\theta_{j,\;m}\left(t\right)\\ \; & \;=\frac{1}{\nu^{\alpha -1}N_{j,m}^{r}N_{m}^{\mu }}\left(\frac{d^{\alpha }}{dt^{\alpha }}+\frac{1}{\tau_{q}\nu^{\alpha -1}}\right)\sum_{i=1}^{n}\int_{r_{i-1}}^{r_{i}}r^{2}R_{i,\;j,\;m}^{\Lambda }\left(r\right)\left(\int_{\mu_{\varphi }}^{1}g_{i}\left(r,\mu ,t\right)P_{\beta_{m}}\left(\mu \right)d\mu \right)dr. \end{array}$$

This differential equation is complemented by initial conditions which are derived using Equations (19), (20) and (59) and the orthogonality conditions (43) and (56). The initial conditions for the function $\theta_{j,\;m}$ have the form

(61) $${\hat{\theta }}_{j,m}^{0}\equiv \theta_{j,\;m}\left(0\right)=\frac{1}{N_{j,m}^{r}}\sum_{i=1}^{n}\frac{\lambda_{i}}{\kappa_{i}}\int_{r_{i-1}}^{r_{i}}r^{2}R_{i,\;j,m}^{\Lambda }\left(r\right)\left[\frac{1}{N_{m}^{\mu }}\int_{\mu_{\varphi }}^{1}F_{i}\left(r,\mu \right)P_{\beta_{m}}\left(\mu \right)d\mu -R_{i,m}^{\psi }\left(r\right)\right]dr,$$

(62) $${\hat{\theta }}_{j,m}^{\alpha }\equiv {\left.\frac{\partial^{\alpha }}{\partial t^{\alpha }}\theta_{j,\;m}\left(t\right)\right|}_{t=0}=\frac{1}{N_{j,m}^{r}N_{m}^{\mu }}\sum_{i=1}^{n}\frac{\lambda_{i}}{\kappa_{i}}\int_{r_{i-1}}^{r_{i}}r^{2}R_{i,\;j^{\prime },m}^{\Lambda }\left(r\right)\int_{\mu_{\varphi }}^{1}G_{i}\left(r,\mu \right)P_{\beta_{m}}\left(\mu \right)d\mu dr.$$

In order to determine a solution of the initial value problem (60) and (61), we apply the Laplace transform technique. The Laplace transform $\bar{f}\left(s\right)$ of the function $f\left(t\right)$ is defined as

(63) $$\bar{f}\left(s\right)=L\left[f\left(t\right)\right]=\int_{0}^{\infty }f\left(t\right)e^{-st}dt.$$

The Laplace transform of the Caputo derivative of order $\alpha \in \left(0,1\right]$ is given by

(64) LDtα0Cft=sαf¯s−sα−1f0.

The application of the Laplace transform to Equation (60) allows us to express the transform ${\bar{\theta }}_{j,m}\left(s\right)$ in the form

(65) $$\begin{array}{cc} {\bar{\theta }}_{j,m}\left(s\right)= & \frac{1}{s}\left(1-\frac{\omega_{j,m}^{2}}{\tau_{q}\nu^{2\alpha -2}}{\bar{K}}_{j,m}\left(s\right)\right){\hat{\theta }}_{j,m}^{0}+s^{\alpha -1}{\bar{K}}_{j,m}\left(s\right){\hat{\theta }}_{j,m}^{\alpha }\\ \; & +\frac{1}{\nu^{\alpha -1}N_{j,m}^{r}N_{m}^{\mu }}\left(s^{\alpha }+\frac{1}{\tau_{q}\nu^{\alpha -1}}\right){\bar{K}}_{j,m}\left(s\right)\sum_{i=1}^{n}\frac{\lambda_{i}}{\kappa_{i}}\int_{r_{i-1}}^{r_{i}}\int_{\mu_{\varphi }}^{1}r^{2}R_{i,j,m}^{\Lambda }\left(r\right)P_{\beta_{m}}\left(\mu \right){\bar{g}}_{i}\left(r,\mu ,s\right)d\mu \;dr\\ \; & -\frac{1}{\nu^{\alpha -1}N_{j,m}^{r}N_{m}^{\mu }}s^{\alpha -1}{\bar{K}}_{j,m}\left(s\right)\sum_{i=1}^{n}\frac{\lambda_{i}}{\kappa_{i}}\int_{r_{i-1}}^{r_{i}}\int_{\mu_{\varphi }}^{1}r^{2}R_{i,j,m}^{\Lambda }\left(r\right)P_{\beta_{m}}\left(\mu \right)g_{i}\left(r,\mu ,0\right)d\mu dr, \end{array}$$

where

(66) $${\bar{K}}_{j,m}\left(s\right)={\left[s^{2\alpha }+\frac{1}{\tau_{q}\nu^{\alpha -1}}\left(\left(1+\tau_{T}\;\omega_{j,\;m}^{2}\right)s^{\alpha }+\frac{\omega_{j,\;m}^{2}}{\tau_{q}\nu^{2\alpha -2}}\right)\right]}^{-1}.$$

For the purpose of determining the inverse Laplace transform $L^{-1}\left[{\bar{\theta }}_{j,\;m}\left(s\right)\right]$, we find firstly four inverse transforms: $K_{j,\;m}\left(t\right)=L^{-1}\left[{\bar{K}}_{j,\;m}\left(s\right)\right]$, $K_{j,m}^{*}\left(t\right)=L^{-1}\left[s^{\alpha }{\bar{K}}_{j,\;m}\left(s\right)\right]$, $K_{j,m}^{**}\left(t\right)=L^{-1}\left[s^{-1}{\bar{K}}_{j,\;m}\left(s\right)\right]$ and $K_{j,m}^{***}\left(t\right)=L^{-1}\left[s^{\alpha -1}{\bar{K}}_{j,\;m}\left(s\right)\right]$. Introducing notation: $\vartheta_{j,\;m}=p_{j,\;m}^{2}-q_{j,\;m}$ with $p_{j,\;m}=\frac{1}{2\tau_{q}\nu^{\alpha -1}}\left(1+\tau_{T}\omega_{j,\;m}^{2}\right)$ and $q_{j,\;m}=\frac{\omega_{j,\;m}^{2}}{\tau_{q}\nu^{2\left(\alpha -1\right)}}$, we rewrite ${\bar{K}}_{j,\;m}\left(s\right)$ as

(67) $${\bar{K}}_{j,m}\left(s\right)=\frac{1}{2\;\sqrt{\vartheta_{j,\;m}}}\left(\frac{1}{s^{\alpha }+z_{j,m}^{-}}-\frac{1}{s^{\alpha }+z_{j,m}^{+}}\right),$$

where $z_{j,\;m}^{\pm }=p_{j,\;m}\pm \sqrt{\vartheta_{j,\;m}}$. Employing the Laplace transform pair given by

(68) $$L\left[t^{\beta -1}E_{\alpha ,\;\beta }^{\gamma }\left(-z\;t^{\alpha }\right)\right]=\frac{s^{\alpha \;\gamma -\beta }}{{\left(s^{\alpha }+z\right)}^{\gamma }},$$

where $E_{\alpha ,\;\beta }^{\gamma }$ is a three parameter Mittag–Leffler function, which is also known as the Prabhakar function [28], and we obtain the inverse transform $K_{j,m}\left(t\right)=L^{-1}\left[{\bar{K}}_{j,\;m}\left(s\right)\right]$ for $\vartheta_{j,\;m}\neq 0$ in the form

(69) $$K_{j,m}\left(t\right)=\frac{t^{\alpha -1}}{2\sqrt{\vartheta_{j,\;m}}}\;\left(E_{\alpha ,\;\alpha }\left(-z_{j,m}^{-}\;t^{\alpha }\right)-E_{\alpha ,\;\alpha }\left(-z_{j,m}^{+}\;t^{\alpha }\right)\right).$$

If $\vartheta_{j,\;m}=0$, then on the basis of (67), we have

(70) $$K_{j,m}\left(t\right)=\;t^{2\alpha -1}E_{\alpha ,\;2\alpha }^{2}\left(-p_{j,m}\;t^{\alpha }\right).$$

Similarly, we receive

(71) $$\begin{array}{cc} K_{j,m}^{*}\left(t\right) & =L^{-1}\left[s^{\alpha }{\bar{K}}_{j,m}\left(s\right)\right]\\ \; & =\left\{\begin{array}{cc} \frac{t^{\alpha -1}}{2\sqrt{\vartheta_{j,\;m}}}\left(z_{j,m}^{+}E_{\alpha ,\;\alpha }\left(-z_{j,m}^{+}t^{\alpha }\right)-z_{j,m}^{-}E_{\alpha ,\;\alpha }\left(-z_{j,m}^{-}t^{\alpha }\right)\right), & \;\vartheta_{j,m}\neq 0,\\ t^{\alpha -1}E_{\alpha ,\;\alpha }^{2}\left(-p_{j,m}t^{\alpha }\right), & \;\vartheta_{j,m}=0. \end{array}\right. \end{array}$$

Using the property of the Laplace transforms, we can find the inverse Laplace transform $L^{-1}\left[s^{-1}{\bar{K}}_{j,\;m}\left(s\right)\right]$ as an integral of the function $K_{j,m}\left(t\right)$. Utilizing the formula for integration of the Mittag–Leffler and Prabhakar functions

(72) $$\int_{0}^{t}u^{\beta -1}E_{\alpha ,\beta }^{\gamma }\left(z\;u^{\alpha }\right)\;du=t^{\beta }E_{\alpha ,\;\beta +1}^{\gamma }\left(z\;t^{\alpha }\right),$$

in Equations (69) and (70), we obtain

(73) $$\begin{array}{cc} K_{j,m}^{**}\left(t\right) & =L^{-1}\left[\frac{1}{s}{\bar{K}}_{j,m}\left(s\right)\right]\\ \; & =\left\{\begin{array}{cc} \frac{t^{\alpha }}{2\sqrt{\vartheta_{j,\;m}}}\left(E_{\alpha ,\;\alpha +1}\left(-z_{j,m}^{-}t^{\alpha }\right)-E_{\alpha ,\;\alpha +1}\left(-z_{j,m}^{+}t^{\alpha }\right)\right), & \vartheta_{j,m}\neq 0,\\ t^{2\alpha }E_{\alpha ,\;2\alpha +1}^{2}\left(-p_{j,m}t^{\alpha }\right), & \vartheta_{j,m}=0. \end{array}\right. \end{array}$$

The inverse Laplace transform $L^{-1}\left[s^{\alpha -1}{\bar{K}}_{j,\;m}\left(s\right)\right]$ can be obtained using Formula (68)

(74) $$\begin{array}{cc} K_{j,m}^{***}\left(t\right) & =L^{-1}\left[s^{\alpha -1}{\bar{K}}_{j,m}\left(s\right)\right]\\ \; & =\left\{\begin{array}{cc} \frac{1}{2\sqrt{\vartheta_{j,\;m}}}\left(E_{\alpha ,1}\left(-z_{j,m}^{-}t^{\alpha }\right)-E_{\alpha ,1}\left(-z_{j,m}^{+}t^{\alpha }\right)\right), & \vartheta_{j,m}\neq 0,\\ E_{\alpha ,1}^{2}\left(-p_{j,m}t^{\alpha }\right), & \vartheta_{j,m}=0. \end{array}\right. \end{array}$$

Employing the functions $K_{j,m}$, $K_{j,m}^{*}$, $K_{j,m}^{**}$ and the Laplace transform ${\bar{\theta }}_{j,\;m}$ given by Equation (65), we can write the functions $\theta_{j,\;m}$ as

(75) $$\begin{array}{cc} \theta_{j,\;m}\left(t\right)= & \left[1-q_{j,\;m}K_{j,\;m}^{**}\left(t\right)\right]{\hat{\theta }}_{j,m}^{0}+K_{j,\;m}^{***}\left(t\right){\hat{\theta }}_{j,m}^{\alpha }\\ \; & +\frac{1}{\nu^{\alpha -1}N_{j,m}^{r}N_{m}^{\mu }}\sum_{i=1}^{n}\frac{\lambda_{i}}{\kappa_{i}}\int_{r_{i-1}}^{r_{i}}\int_{\mu_{\varphi }}^{1}r^{2}R_{i,\;j,m}^{\Lambda }\left(r\right)P_{\beta_{m}}\left(\mu \right)\int_{0}^{t}\left(K_{j,\;m}^{*}\left(\tau \right)+\frac{K_{j,m}\left(\tau \right)}{\tau_{q}\nu^{\alpha -1}}\right)g_{i}\left(r,\mu ,t-\tau \right)\;d\tau \;d\mu \;dr\\ \; & -\frac{1}{\nu^{\alpha -1}N_{j,m}^{r}N_{m}^{\mu }}K_{j,\;m}^{***}\left(t\right)\sum_{i=1}^{n}\frac{\lambda_{i}}{\kappa_{i}}\int_{r_{i-1}}^{r_{i}}\int_{\mu_{\varphi }}^{1}r^{2}R_{i,\;j,m}^{\Lambda }\left(r\right)P_{\beta_{m}}\left(\mu \right)g_{i}\left(r,\mu ,0\right)d\mu \;dr. \end{array}$$

If the functions $g_{i}$ are given by

(76) $$g_{i}\left(r,\mu ,t\right)=\left\{\begin{array}{cc} Q_{1} & \;\mathrm{for}\;\;i=1,\\ 0 & \;\mathrm{for}\;\;i=2,\;\ldots ,\;n. \end{array}\right.$$

where $Q_{1}$ is a constant, then the functions $\theta_{j,\;m}$ can be written in the form

(77) $$\begin{array}{cc} \theta_{j,\;m}\left(t\right)= & \left(1-q_{j,m}K_{j,m}^{**}\left(t\right)\right){\hat{\theta }}_{j,m}^{0}\\ \; & +\frac{\lambda_{1}Q_{1}\delta_{m,\;0}}{\tau_{q}\nu^{2\alpha -2}\kappa_{1}N_{j,0}^{r}\Omega_{1,\;j,\;0}^{3}}\left(\sin\left(\Omega_{1,\;j,\;0}r_{1}\right)-\Omega_{1,\;j,\;0}r_{1}\cos\left(\Omega_{1,\;j,\;0}r_{1}\right)\right)K_{j,0}^{**}\left(t\right). \end{array}$$

Taking the function $\theta_{j,\;m}$ into account in Equation (59), we obtain the function $\phi_{i}$. This function and the function $\psi_{i}$ given by Equation (47) are used in Equation (21), which determines the temperature $T_{i}$ in the i-th layer of the body under consideration.

## 4. Numerical Examples and Discussion

The presented analytical solution of the fractional heat conduction problem was used to compute the temperature distributions in a layered spherical cone to investigate the effect of the phase-lags and the order of the fractional time-derivative on the heat conduction in the medium. The analysis concerns the heat conduction in the cone of constant initial temperature with an inner or outer heat source. We introduce the non-dimensional time $\bar{t}=t\;\kappa_{n}/b^{2}$, the non-dimensional radii $\bar{r}=r/b$ and ${\bar{r}}_{i}=r_{i}/b$ where $r_{i}$ determines the boundary of the i-th spherical layer, i=1,…,n. Temperature T in the cone is defined as $T\left(\bar{r},\mu ,\bar{t}\right)=T_{i}\left(r,\mu ,t\right)$ where $r=\bar{r}\;b$, $t=\bar{t}\;b^{2}/\kappa_{n}$ and i is the layer number; i.e., the condition $r_{i-1}<r\leq r_{i}$ is fulfilled. The spherical cone under consideration consists of five concentric layers wherein ${\bar{r}}_{i}=0.2\;i$, i=1,…,5. The physical data assumed in the computation are given in Table 1.

The role of the fractional order occurring in the mathematical model of the fractional heat conduction and its effect on the temperature distribution in the spherical cone was investigated for $\alpha \in \left[0.4;0.6\right]$. Computer simulation data were generated by the Mathematica software.

**Figure 2:**
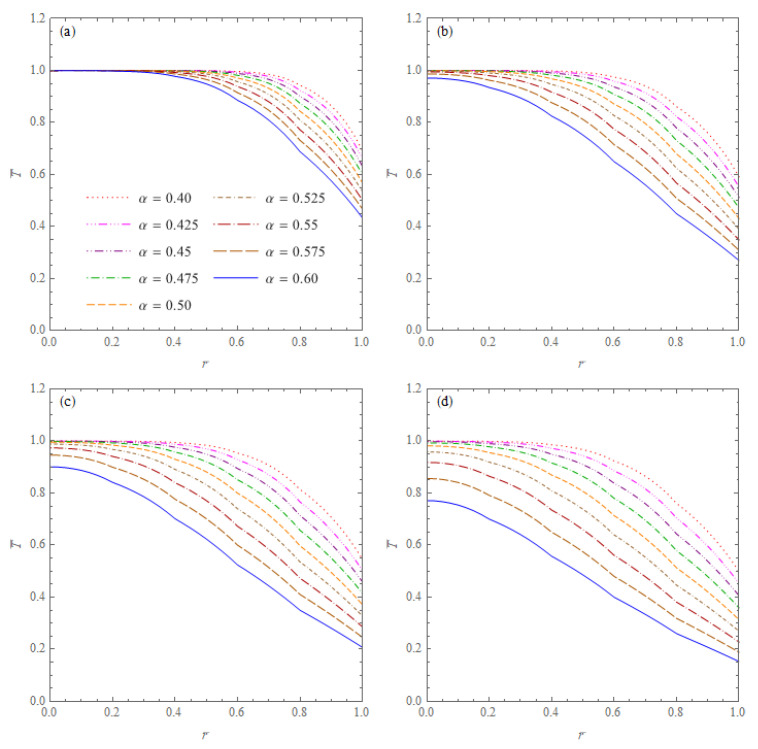
Dimensionless temperatures $\bar{T}=T/T_{0}$ in the cone without inner and outer heat sources with initial temperature $T_{0}$ = 20 °C versus $\bar{r}=r/b$ for six values of the order of fractional derivative α and four values of the non-dimensional time: (**a**) $\bar{t}=1$; (**b**) $\bar{t}=5$; (**c**) $\bar{t}=10$; (**d**) $\bar{t}=20$.

The first example concerns the heat conduction in a spherical cone without inner and outer heat sources. The cone is specified by the radius b=0.5 m and the half-angle at the apex of the cone $\bar{\varphi }=\pi /3$. The initial temperature, the ambient temperature and the heat transfer coefficient were assumed as: *F_i_*(*r*,*μ*) = *T*_0_ = 20 °C, *T_a_* = 0 °C and $a_{\infty }=1200\;W/\left(m^{2}K\right)$, respectively. The computations were performed for the phase lags $\tau_{q}=5$ s, $\tau_{T}=1$ s (artificial values, identical for each layer) and six different values of the order of fractional derivative: α = 0.4; 0.425; 0.45; 0.475; 0.5; 0.525; 0.55; 0.575; 0.6. Figure 2 presents the dimensionless temperatures in the cone $\bar{T}=T/T_{0}$ versus $\bar{r}=r/b$ for the non-dimensional time variable $\bar{t}=1;5;10;20$. The results show the effect of the order of fractional derivative occurring in the heat conduction equation on the temperature distribution in the cone. The temperatures in the points of the cone tend with time to a steady state for each value of the derivative order α. It can be noted that temperature decreases faster for larger values of the order α occurring in the mathematical model of the heat conduction.

In the case of the heat conduction in the spherical cone without an inner heat source, we predict that the function $\phi_{i}$ occurring in Equation (21) satisfies the condition

(78) $$\lim_{t\to \infty }\phi_{i}\left(r,\mu ,t\right)=0,$$

for i=1,…,n and all $r\in \left[0,b\right]$ and $\mu \in \left[\mu_{\varphi },1\right)$, i.e., the steady state of temperature distribution: $T_{i}\left(r,\mu ,t\right)=\psi_{i}\left(r,\mu \right)$ occurs in the cone. For the purpose of the formal proof of this statement, the asymptotic formula may be used for the Mittag–Leffler function $E_{\alpha ,\beta }$ given by

(79) $$E_{\alpha ,\beta }\left(z\right)=-\sum_{k=1}^{m}\frac{z^{-k}}{\Gamma \left(\beta -k\alpha \right)}+O\left({\left|z\right|}^{-m-1}\right),\;\;\;\;\;\left|z\right|\to \infty .$$

**Figure 3:**
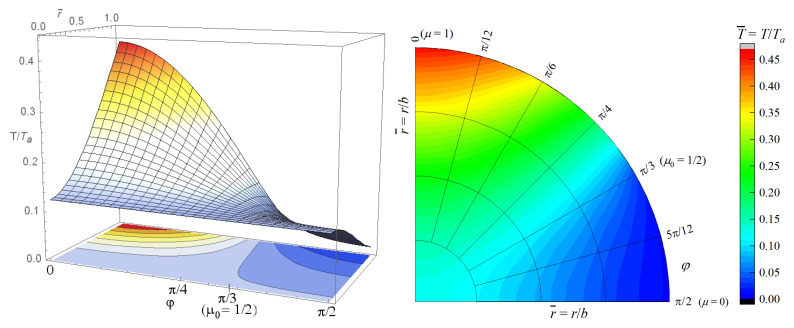
Temperature distribution $T/T_{a}$ in the hemisphere without an inner heat source when its surface is heated by an outer heat source defined by Equation (50) with $\mu_{0}=1/2$ and $T_{a}$ = 40 °C , as a function of non-dimensional space variables $\left(\bar{r},\varphi \right)$.

Employing this formula in Equation (72), we obtain that $\lim_{t\to \infty }K_{j,m}^{**}\left(t\right)=1/q_{j,m}$. Taking into account the first term on the right-hand side of Equation (76) for consideration of the heat conduction without an inner source, we have $\lim_{t\to \infty }\theta_{j,\;m}\left(t\right)=0$ for j=1,2,… and m=0,1,2,… Hence, on the basis of Equation (59), the condition (78) is fulfilled. Thus, the steady-state temperature distribution in the i-th layer of the solid sphere is given by Equation (21) in which the first term on the right-hand side can be omitted. In this case, the temperature in the considered region is independent from the phase-lags and the order of fractional derivatives which occur in the heat conduction model. In Figure 3, the 3D graph and contour plot of the function $T_{i}=\psi_{i}\left(r,\mu \right)$ illustrating the change of temperature in a hemisphere when its surface is heated by an outer heat source defined by Equation (50) with $\mu_{0}=1/2$ and $T_{a}$ = 40 °C shown. The physical data that characterized the hemisphere, which were used in computation, are the same as in the first example.

**Figure 4:**
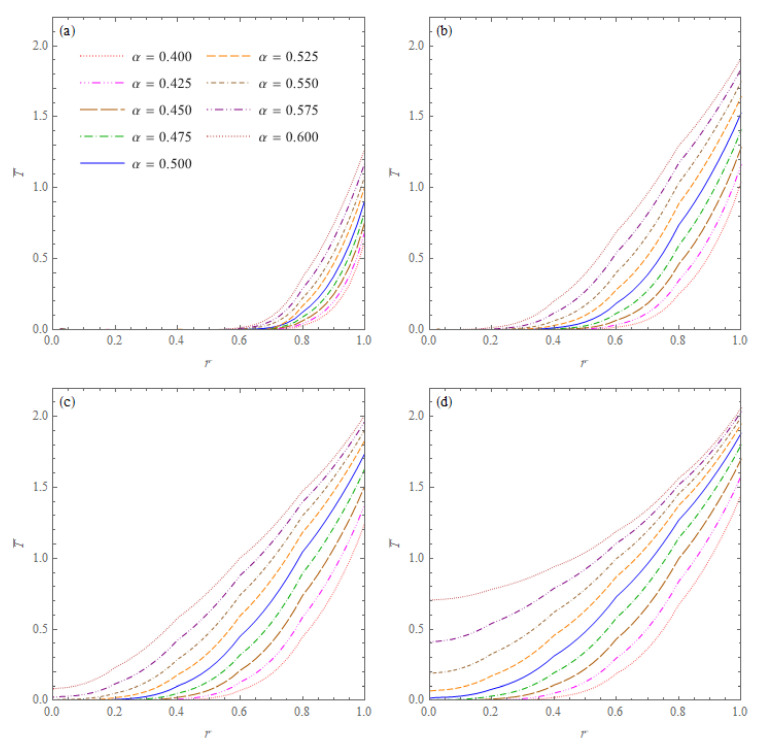
Dimensionless temperatures $\bar{T}$= *T*/20 °C in the cone without inner heat sources with initial temperature $T_{0}$ = 0 °C , versus $\bar{r}=r/b$ for six values of the order of fractional derivative α and four values of the non-dimensional time: (**a**) $\bar{t}=0.05$; (**b**) $\bar{t}=0.25$; (**c**) $\bar{t}=0.5$; (**d**) $\bar{t}=1$.

The Robin condition (18) on the spherical surface of the cone describes the heat exchange with the environment. If the ambient temperature is higher than the temperature of the spherical surface of the cone, then the spherical surface is heated, and the heat flows in the cone in the radial direction. In Figure 4, curves are presented that illustrate the changes of temperatures in the cone when the ambient temperature is specified by Equation (50) with $T_{0}$ = 0 °C and $T_{a}$ = 20 °C. The calculations were performed assuming that there is no internal heat source.

**Figure 5:**
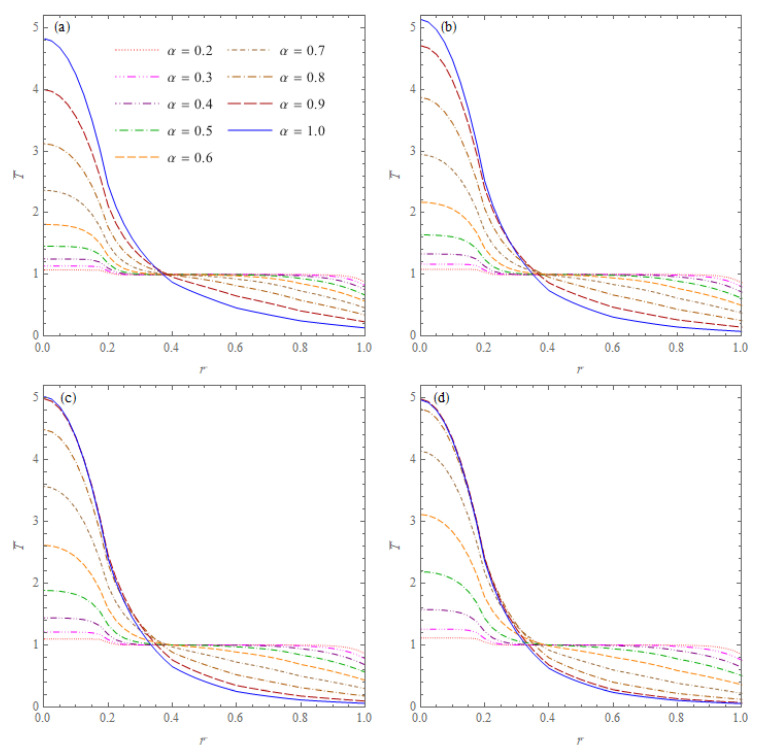
Dimensionless temperatures $\bar{T}$ = *T*/20 °C in the cone with an inner heat source described by Equation (76) with $Q_{1}=2\times 10^{6}$ $W/m^{3}$, versus $\bar{r}=r/b$ for six values of the order of fractional derivative α and four values of the non-dimensional time: (**a**) $\bar{t}=0.25$; (**b**) $\bar{t}=0.5$; (**c**) $\bar{t}=1$; (**d**) $\bar{t}=1.5$.

The next example concerns the spherical cone heated by the internal heat source described by Equation (76). The curves in Figure 5 present temperature distributions in the spherical cone for volumetric heat source $Q_{1}=2\times 10^{6}$ $W/m^{3}$ and four values of dimensionless time. Decreasing the temperature while increasing the radial distance from the heat source follows as a result of the heat exchange through the spherical surface of the cone with the environment of zero temperature. It should be noted that the temperatures in a part of the cone close to the heat source are higher for the higher fractional orders of the heat conduction models, and the temperatures in a part of the cone close to the spherical surface are higher for the lower orders of the conduction models.

## 5. Conclusions

The analytical solution of the heat conduction problem for a spherical cone with an internal and external heat source is presented. The problem formulation and its solution include also the special cases for the hemisphere and solid sphere. The fractional heat conduction model with a Caputo time-derivative includes phase-lags of the temperature and heat flux. The solution in the form of a double series of spherical Bessel functions and Legendre functions was derived. Numerical investigation of the effect of the fractional order of the time derivative occurring in the heat conduction equation is presented. According to the mathematical model, the fractional order of the differential equation has significant importance for temperature distribution in the spherical cone. It was stated that the higher order of fractional derivative in the heat conduction model leads to the higher temperature in the heated body and lower temperature in the cooled body. As was expected, for each value of the derivative order α, the temperature in all points of the cone tends with time to a steady state. However, as can be seen in presented examples, the temperature decreases/increases faster for larger values of the order occurring in the mathematical model of the fractional heat conduction.

In the future, we would like to develop a numerical model for solving the considered problem and make a comparative analysis of the obtained results. We think that in the case of a numerical solution, the constant values of parameters in the considered model can be replaced by functional dependencies, e.g., the temperature-dependent thermophysical parameters of materials in each layer. In addition, in the future research, we would like to include, among others, an external moving heat source in the heat conduction model presented in this paper and find analytical and/or numerical solutions for them. We would like to find the practical applications of the considered fractional heat conduction model, e.g., in modeling the influence of solar energy on the heating of the spherical cone [40].

## Figures and Tables

**Table 1:** Thermal diffusivity and thermal conductivity of the spherical layers of the cone applied in the numerical examples.

*i*	1	2	3	4	5
κim2m2ss	$3.3\times 10^{-6}$	$6.0\times 10^{-6}$	$1.1\times 10^{-5}$	$2.0\times 10^{-5}$	$4.0\times 10^{-5}$
λiWWmKmK	16.0	24.0	36.0	54.0	81.0

## Data Availability

Not applicable.
